# Analysis of the Expression and Function of Immunoglobulin-Like Transcript 4 (ILT4, LILRB2) in Dendritic Cells from Patients with Systemic Lupus Erythematosus

**DOI:** 10.1155/2016/4163094

**Published:** 2016-02-22

**Authors:** Paola del Carmen Guerra-de Blas, Yael Sebastián Villaseñor-Talavera, Daniela de Jesús Cruz-González, Lourdes Baranda, Lesly Doníz-Padilla, Carlos Abud-Mendoza, Roberto González-Amaro, Adriana Elizabeth Monsiváis-Urenda

**Affiliations:** ^1^Departamento de Inmunología, Facultad de Medicina, Universidad Autónoma de San Luis Potosí, Avenida Venustiano Carranza 2405, 78210 San Luis Potosí, SLP, Mexico; ^2^Departamento de Reumatología, Hospital Central “Dr. Ignacio Morones Prieto”, Avenida Venustiano Carranza 2395, 78290 San Luis Potosí, SLP, Mexico

## Abstract

Dendritic cells (DC) play an important role in the development and maintenance of immune tolerance. Although the inhibitory receptor ILT4/LILRB2 has been related with the tolerogenic phenotype of DC, the possible role of this receptor in the breakdown of DC tolerogenic function in systemic lupus erythematosus (SLE) has not been elucidated. In this study, we analyzed the expression and function of the inhibitory receptor ILT4 in DC from SLE patients. We found that the percentage of ILT4 positive plasmacytoid DC and myeloid DC is significantly diminished in SLE patients. Interestingly, ligation of ILT4 did not affect the maturation or immunogenic capability of DC in healthy controls. In contrast, in SLE patients we observed an inhibitory effect of ILT4 on the immunogenic capability of DC. ILT4 was shown not to have a crucial role in regulating the maturation and function of DC from healthy controls but is partially involved in the maturation process and immunogenic capability of DC from SLE patients, suggesting that other inhibitory receptors, involved in the regulation of DC tolerogenic function, may be impaired in this autoimmune disease.

## 1. Introduction

Dendritic cells (DC) are a subpopulation of leukocytes specialized in the capture and process of antigens and its presentation to T lymphocytes. In their immature state, they are located in peripheral tissues acting as sentinels. Tissue residing DC form a close network, optimally positioned to sense invading pathogens. The antigens taken up by DC in the periphery are efficiently transported to T cell areas of local lymph nodes. Upon stimulation, DC undergo maturation characterized by the expression of high levels of MHC II and costimulatory molecules, leading to robust T cell activation [1].

Human blood DC are broadly defined as HLA-DR positive leukocytes lacking expression of specific markers for T cell, B cell, NK cell, monocyte, and granulocyte lineages. They can be subdivided into the CD11c^−^ plasmacytoid DC population, which also express CD123, CD303 (BDCA2), and CD304 (BDCA4); and CD11c^+^ CD1c (BDCA1) myeloid DC subset [2].

It is well known that DC are critical regulators of the immune response and clues in the maintenance of peripheral and central tolerance [3]. Tolerogenic properties of DC depend on their maturation state, exposure to anti-inflammatory and immunosuppressive agents, the nature of the microbial stimuli, and environmental cues from the tissue microenvironment, as well as receptors expressed on their cell surface [4, 5]. In this regard, several reports demonstrate that the expressions of the inhibitory molecules IDO, PDL, and ICOSL and receptors of the ILT family (Ig-like transcripts) play a central role in conferring a tolerogenic state on DC [6–8].

Ig-like transcripts (ILTs), also called lymphocyte inhibitory receptors or leukocyte immunoglobulin- (Ig-) like receptors (LIR/LILRs) that correspond to CD85, are a group of membrane receptors coded by more than 10 genes located in the 19q13.4 chromosome. The ILT family receptors are composed of active and inhibitory members. Inhibitory LILRs transmit signals through their long cytoplasmic tails, which contain between two and four immunoreceptor tyrosine-based inhibitory domains (ITIMs) that, upon phosphorylation, recruit SHP-1 and SHP-2 phosphatases, which are involved in the inhibition of different intracellular signal pathways [9]. The best-characterized inhibitory receptors are ILT2 (LILRB1), ILT3 (LILRB4), and ILT4 (LIRB2). ILT4 is expressed mainly by monocytes, macrophages, and dendritic cells. ILT4 ligands are class I HLA molecules. Like the other inhibitory members of the ILT family, ILT4 recruits SHP-1 protein tyrosine phosphatases and mediates a negative signal that inhibits early signaling events [10].

ILT4 modulates several antigen-presenting functions mediated by myelomonocytic cells, such as cytokine production and costimulatory function, and it can also inhibit the activating signal triggered by Fc receptors [11]. It is also known that the continuous ligation of ILT2 and ILT4 inhibits DC differentiation and maturation [12, 13].

Emerging data demonstrate that immunosuppressive factors, like IL-10 and vitamin D, as well as T suppressor lymphocytes, induce the upregulation of ILT4 [12, 14]. DC expressing high levels of ILT3 and ILT4 cocultured with tetramers of soluble HLA-G showed an impaired upregulation of the costimulatory proteins CD80 and CD86 [15]. Thus, HLA-G-ILT interaction leads to the development of tolerogenic DC with the consequent induction of anergic and immunosuppressive T cells [16]. Furthermore, DC expressing higher levels of ILT4 are able to induce regulatory T cells [17].

Inhibitory receptors, such as ILT2 and ILT4, are involved in the tolerogenic effect of DC and previous studies have indicated the important role of these receptors in the pathogenesis of autoimmune diseases [18–20]. SLE, the prototype of autoimmune diseases, is a chronic systemic autoimmune disease. Previous studies have shown that ILT2 may have a role in the pathogenesis of SLE [21–23]. We have demonstrated that peripheral blood mononuclear cells (PBMCs) isolated from patients with SLE exhibit an impaired ILT2 function, whereas B cells express low levels of this receptor [23]. Regarding DC, it has been proposed that pDC play a pivotal role in the development of SLE. The impaired clearance of apoptotic cells observed in SLE and the opsonization of cellular apoptotic debris by autoantibodies enhances its uptake by pDC. Moreover, these apoptotic particles are able to induce the synthesis of IFN-*α*, which in turn favors the maturation of mDC and triggers isotype switching and autoantibody production by autoreactive B cells [24–26]. However, the role of ILT4 in the pathophysiology of this autoimmune disease has not been elucidated.

The aim of this work was to study the expression of ILT4 in peripheral blood DC and to study the inhibitory function of this receptor in monocyte-derived dendritic cells from SLE and healthy patients.

## 2. Materials and Methods

### 2.1. Patients

Thirty-four patients (32 female and 2 male) with diagnosis of SLE were included. Diagnosis was made according to the classification criteria of the American College of Rheumatology [27]. Mean age was 35.2 years, and mean duration of disease was 4.47 years at the time of the study. Twelve patients had active disease at the time of the study. Disease activity was scored according to the SLEDAI index [28] and the arithmetic mean of SLEDAI was 5.96. All our patients were receiving immunosuppressive drugs at the time of the study. The drugs included prednisone, hydroxychloroquine, azathioprine, mycophenolate mofetil, and cyclosporine, alone or in combination. All clinical characteristics are summarized in Table 1. Thirty-four healthy individuals with similar age and same sex compared to patients were included as controls. In all cases, an informed written consent was obtained, and the local Ethics Committee (Ethics and Research Committee of the Hospital Central Dr. Ignacio Morones Prieto) approved this study. This work was carried out in accordance with The Code of Ethics of the World Medical Association (Declaration of Helsinki) for experiments involving humans.

### 2.2. Cell Isolation and DC Generation

Peripheral blood mononuclear cells (PBMCs) were isolated by Ficoll-Hypaque (Sigma Chemical Co., St. Louis, MO) centrifugation. To isolate monocytes, PBMCs were incubated with anti-CD14 mAb coated microbeads followed by positive selection using MACS single-use separation columns from Miltenyi Biotec (Bergisch Gladbach, Germany). The purity of monocytes was assessed by flow cytometry analysis on the basis of CD14 expression and was always higher than 90%.

For the* in vitro* generation of DC (moDC), purified monocytes at a final concentration of 1 × 10^6^ cells/mL were incubated in RPMI-1640 culture medium (GIBCO, Grand Island, NY) supplemented with 15% heat inactivated fetal bovine serum (FBS), 2 mM glutamine, 100 U/mL penicillin and 100 mg/mL streptomycin, nonessential amino acids and sodium pyruvate, GM-CSF (50 ng/mL), and IL-4 (15 ng/mL) for 6 days. Culture medium and cytokines were replaced every 2 days. In order to evaluate the inhibitory function of ILT4 and its possible synergy with ILT2, DC were cultured for two additional days with LPS (100 ng/mL) to induce maturation at three different conditions: in the presence or absence of the agonist anti-ILT4 purified antibody (10 *μ*g/mL) or in the presence of the agonist anti-ILT4 at the same concentration, plus the agonist anti-ILT2 purified antibody (20 *μ*g/mL) (BioLegend, San Diego, CA). Cells were harvested at days 6 and 9, washed, labeled, and analyzed for the expression of the indicated maturation markers.

### 2.3. Antibodies

The following monoclonal antibodies (mAbs) were used: anti-CD83 labeled with APC, anti-CD80-FITC, anti-CD40-PE, anti-CD86 coupled to PERCP-Cy5, anti-Lin-FITC, anti-HLA-DR-APC-Cy7, anti-CD11c-PerCP-Cy5.5 (BD Biosciences, San Jose, CA), anti-BDCA1 and anti-BDCA4 (Miltenyi Biotech) tagged with APC, and anti-ILT4 labeled with PE (BioLegend, San Diego, CA). For functional studies, purified anti-human ILT4 and purified anti-human ILT2 mAbs were employed (BioLegend, San Diego, CA).

### 2.4. Flow Cytometry Analysis

**Figure 1:**
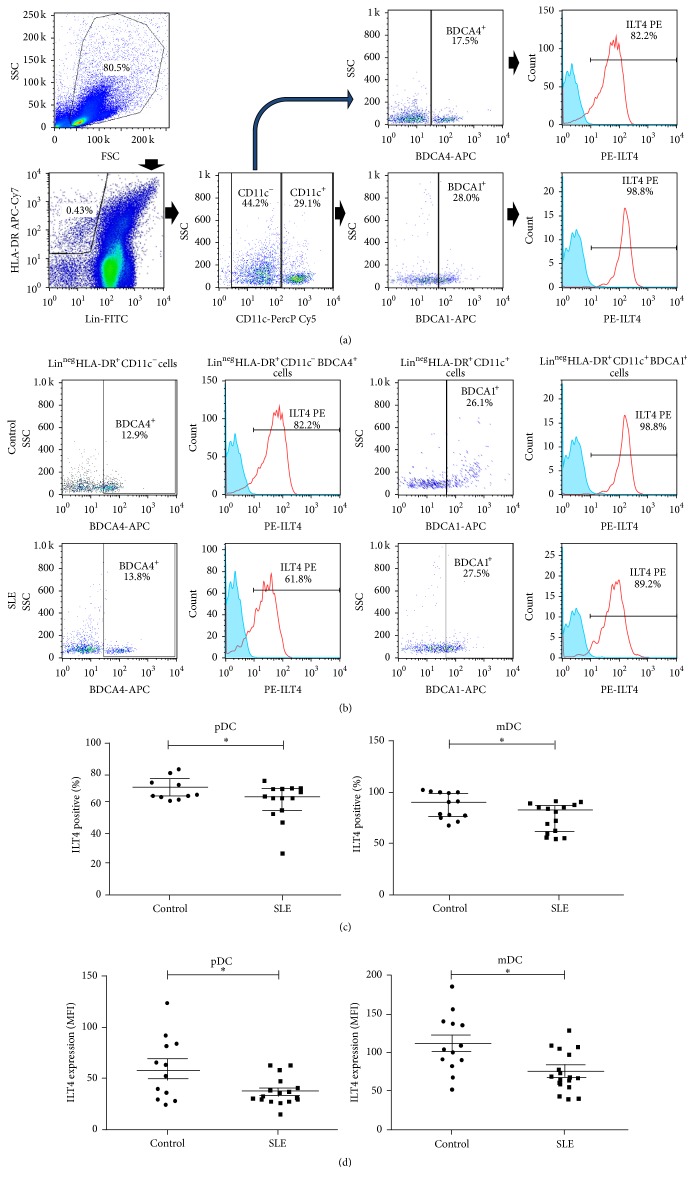
Expression of ILT4 by DC in the peripheral blood from SLE patients and healthy controls. (a) Polychromatic flow cytometry gating tree for lineage-negative (Lin^−^), HLA-DR^+^, CD11c^+^, and BDCA1^+^ cells (mDC) and Lin^−^, HLA-DR^+^, CD11c^−^, and BDCA4^+^ cells (pDC). ILT4 positive cells were evaluated in each subpopulation. Data shown in (b) and (c) were generated based on this gating tree. Cut-offs for background fluorescence were based on isotype-matched Ig negative controls and FMO (Fluorescence Minus One) strategy. (b) PBMCs from SLE patients and healthy subjects included in the study were immunostained for the detection of ILT4 expression on pDC and mDC by flow cytometry, as stated in Section 2. Percentages of ILT4^+^ mDC or ILT4^+^ pDC were calculated for Lin^−^HLA^−^DR^+^CD11c^+^BDCA1^+^ or Lin^−^HLA^−^DR^+^CD11c^−^BDCA4^+^ cells, respectively. Representative histograms from cells of one control and one SLE patient (upper and lower panel, resp.) are shown. Numbers indicate the percentage of BDCA4^+^ cells from Lin^−^HLA-DR^+^CD11c^−^ gate or BDCA1^+^ cells from Lin^−^HLA-DR^+^CD11c^+^ gate (first and third panels) and ILT4^+^ leukocytes from Lin^−^HLA-DR^+^CD11c^−^BDCA4^+^ or Lin^−^HLA-DR^+^CD11c^+^BDCA1^+^ gate (second and fourth panels). (c) Percentages of ILT4 positive pDC (left panel) and mDC (right panel) from SLE patients and healthy controls. ^*∗*^ *p* < 0.05. (d) MFI of ILT4 expression on pDC (left panel) and mDC (right panel).

PBMCs were labeled with 5 *μ*L of a FITC anti-human lineage antibody cocktail (anti-Lin), 7 *μ*L of APC-Cy7 tagged anti-HLA-DR, 5 *μ*L of PerCp-Cy5.5 labeled anti-CD11c, 7 *μ*L of APC labeled anti-BDCA1 or anti-BDCA4, and 5 *μ*L of anti-ILT4 PE, for 20 min at 4°C. Then, cells were washed, fixed with 1% PFA, and analyzed in a FACSAria II cytometer (BD Biosciences), using the FACSDiva and FlowJo software (BD Biosciences). DC derived from monocytes and* in vitro* generated were harvested at days 6 and 9 and labeled with 3 *μ*L of FITC anti-CD80, PerCP-Cy5 anti-CD86, and PE anti-CD40. In all assays, Fc*γ*R receptors were previously blocked with 10% human AB serum. In order to set gates, we used the FMO (Fluorescence Minus One) strategy. In brief, FMO controls leave out one reagent at a time (the opposite of single stain controls). In FMO, a control is defined as changing one condition at a time (Figure 1).

### 2.5. Cytokine Production

Cytokines levels were determined in culture supernatants using the Cytokine Bead Array (CBA) kit for inflammatory cytokines (BD Biosciences). In brief, supernatants from moDC cultures and moDC-T cells cocultures were collected and cytokine levels were quantified according to manufacturer instructions and then analyzed in FACS Canto II (BD Biosciences).

### 2.6. Cell Proliferation Assays

Cell proliferation was assessed by a fluorescent label partition assay and flow cytometry analysis. Briefly, mature moDC were incubated in three different conditions, medium only, anti-ILT4 (10 *μ*g/mL), or anti-ILT4 (10 *μ*g/mL) + anti-ILT2 (20 *μ*g/mL), and then cocultured with allogenic lymphocytes of one healthy donor, in flat-bottomed 96-well plates precoated with a mixture of the anti-CD3 T_3_B mAb (kindly provided by Dr. Sanchez Madrid, Hospital de la Princesa, Spain) and an anti-CD28 mAb (10 *μ*g/mL). Allogenic lymphocytes were previously loaded with 5.0 *μ*M carboxyfluorescein diacetate-succinimidyl-ester (CFDA-SE, Molecular Probes, Eugene, OR), according to manufacturer's instructions. After five days of culture under standard conditions (with 5% CO_2_ at 37°C and 100% humidity), cells were harvested and analyzed by flow cytometry. Cell proliferation was assessed by measuring the corresponding decrease in cell fluorescence by flow cytometry and the percentage of cell proliferation was normalized with the following formula: % cell proliferation = 100 − ((% cells in nonstimulated culture/% cells in stimulated culture) × 100).

### 2.7. Statistical Analysis

Data were analyzed with the GraphPad Prism, 5.01 software. Flow cytometry data were evaluated by using the Mann-Whitney *U* test. When indicated, Kruskal-Wallis test was also performed. Analysis* post hoc* was made using the Dunnett posttest. The analysis of correlations between variables was based on Spearman's rank test; *p* < 0.05 was considered statistically significant.

## 3. Results

### 3.1. ILT4 Expression by Circulating DC from Patients with SLE

To assess the expression of ILT4 by circulating pDC and mDC, we performed multiparametric flow cytometry. We defined pDC as lineage-negative (Lin^−^), HLA-DR^+^, CD11c^−^, BDCA4^+^, and mDC as Lin^−^, HLA-DR^+^, CD11c^+^, and BDCA1^+^ (Figure 1(a)). The following gating strategy was employed for this purpose: from the SSC and FSC dot plot, we analyzed the Lin^−^HLA-DR^+^ cells; for pDC we considered the percentage of CD11c^−^BDCA4^+^ cells, and for mDC the percentage of CD11c^+^BDCA1^+^ was obtained; then, ILT4 positive cells were analyzed in every DC subpopulation (Figure 1(a)). We found that a very high percentage of mDC from healthy controls expressed ILT4; in contrast, mDC from SLE patients showed a significant lower expression of the inhibitory receptor (*p* = 0.018, Figures 1(b) and 1(c)).

Levels of pDC ILT4^+^ from SLE patients showed a high variability, with percentages varying from 8.1% to 68.8%. Interestingly, percentages of ILT4 positive pDC and mDC were lower in SLE group compared with healthy subjects (*p* = 0.019, Figure 1(c)). Consistently, when we evaluated the surface expression of ILT4 (measured as the mean fluorescence intensity, MFI) in mDC and pDC, we observed that SLE patients DC displayed a lower expression of this receptor (Figure 1(d)). Interestingly, no significant association was detected between expression of ILT4 by pDC or mDC and disease activity or immunosuppressive therapy (*p* > 0.05 for all cases, data not shown).

### 3.2. ILT4 Effect on DC Maturation in SLE

**Figure 2:**
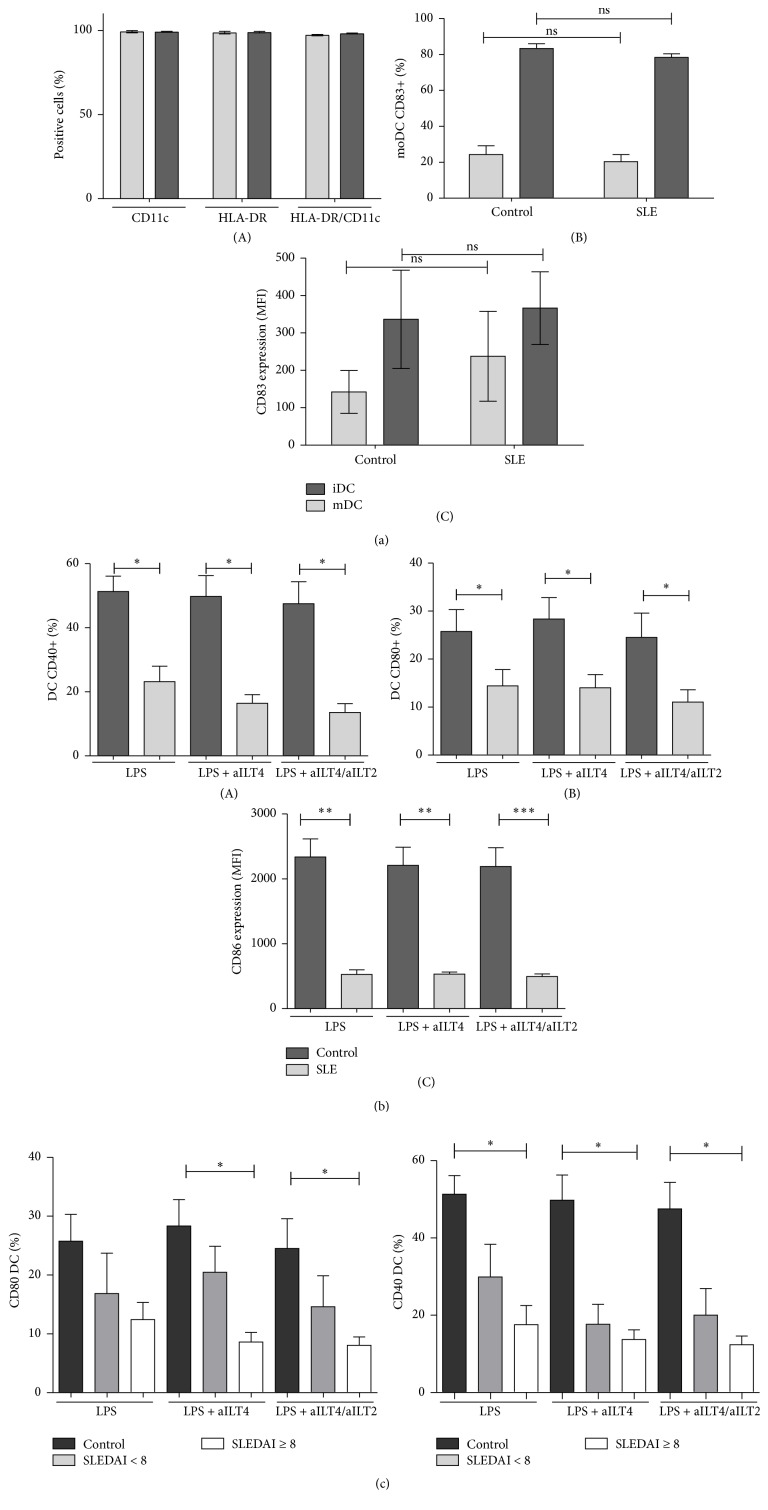
Expression of costimulatory molecules in mature monocyte-derived DC (moDC) from healthy controls compared to SLE patients. Monocytes from SLE patients and healthy controls were induced to differentiate into DC with GM-CSF/IL-4 and then cultured in the presence of LPS in the presence or absence of anti-ILT4 and/or anti-ILT2 agonistics mAbs (aILT4/ILT2). DC were immunostained for CD11c, HLA-DR, CD83, CD80, CD86, and CD40 and analyzed by flow cytometry. (a) CD11c and HLA-DR expression on immature and mature moDC from SLE patients (A). Percentages and MFI of CD83 expression on moDC from SLE patients and healthy controls are shown ((B) and (C)). (b) Data from SLE patients and healthy controls is shown. CD80 and CD40 expression was measured as the percentage of positive cells. CD86 expression was assessed as the mean fluorescence intensity (MFI). (c) Results from SLE patients were grouped according to SLEDAI index score (<8 and ≥8) and compared to healthy controls. ^*∗*^ *p* < 0.05, ^*∗∗*^ *p* < 0.01, and ^*∗∗∗*^ *p* < 0.001.

In the first place, we assessed the phenotype of DC differentiated from monocytes of SLE and healthy subjects by addressing the expression of CD11c, HLA-DR, and CD83. We observed that DC from SLE patients express the same levels of these differentiation markers, and we could not find significant differences between the expression of CD83 on DC from SLE or healthy controls (Figure 2(a)). moDC from SLE patients have been reported to display abnormal responses to different activation stimuli (LPS, TNF-*α*, PGE_2_, or anti-CD40) compared to DC differentiated from healthy monocytes [29]. Consistent with these reports, we observed an abnormal response of moDC to LPS, characterized by a low induction of the costimulatory molecule CD40, analyzed as percentage of CD40 positive cells (Figure 2(b)(A)) as well as by MFI (data not shown). We also detected a diminished expression of CD80 (Figure 2(b)(B)) in response to LPS in SLE moDC compared with healthy controls. CD86 expression in response to the activating stimulus LPS was abnormal too; moDC from SLE subjects showed diminished levels of surface expression of this costimulatory molecule in comparison to control moDC (Figure 2(b)(C)). Disease activity worsened the response to LPS in SLE moDC; moDC isolated from patients with a higher SLEDAI index (≥8) showed lower expression levels of costimulatory molecules (CD80 and CD40) (Figure 2(c)).

**Figure 3:**
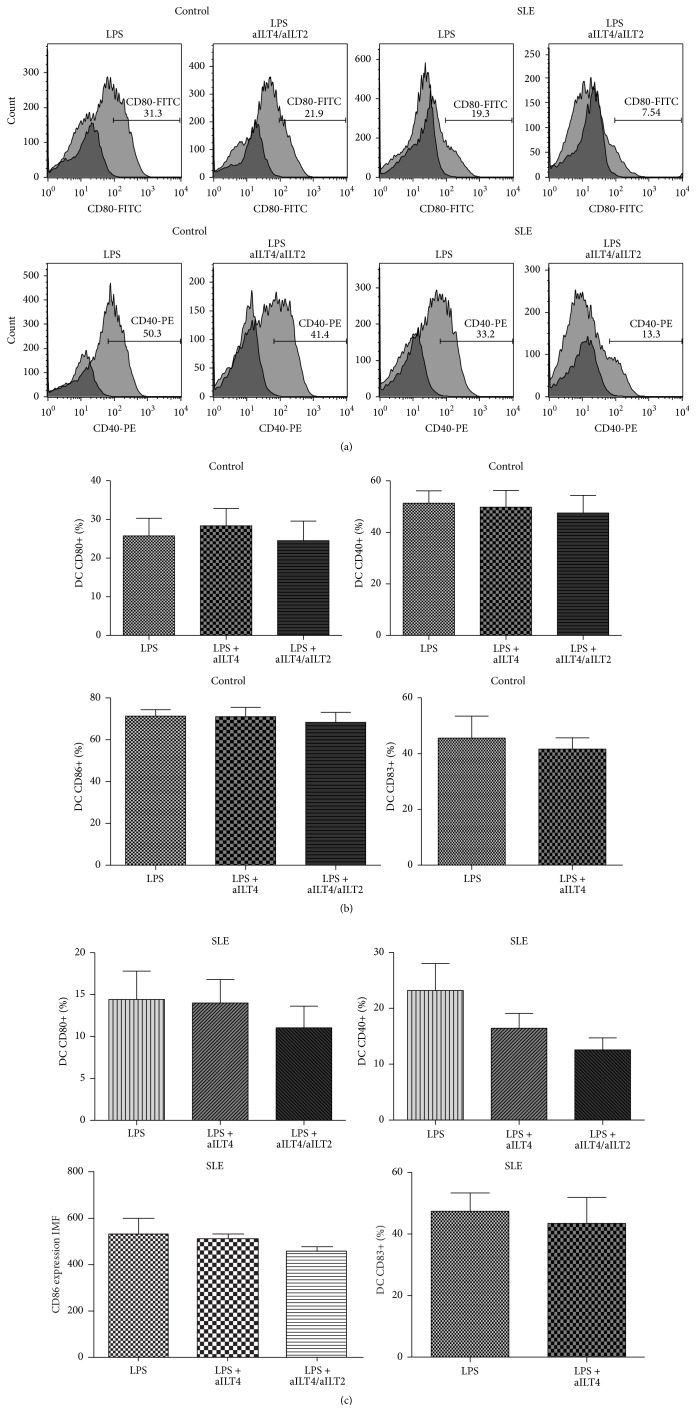
Effect of ILT4 on the expression of costimulatory molecules by mature moDC from healthy controls and SLE patients. (a) Representative dot plots for the expression of CD80 and CD40 in healthy controls (left panel) and SLE patients (right panel). Numbers represent the percentage of CD80 and CD40 positive cells. (b) Analysis of the expression of maturation markers in mature DC from healthy controls and SLE patients, in response to continuous ligation of ILT4 or ILT4/ILT2. Results are represented as the median and the interquartile range.

It has been described that ILT2 ligation inhibits DC maturation [30]; however, a previous study performed in our laboratory showed that ILT2 poorly regulates moDC maturation [22]. Thus, in order to evaluate the possible participation of ILT4 receptor and its synergy with ILT2, we induced moDC maturation in the presence or absence of the anti-ILT4 and/or anti-ILT2 agonist mAb. Unexpectedly we did not find an apparent influence of ILT4 in the maturation of moDC in healthy controls (Figure 3(a)). CD40, CD83, CD80, and CD86 expression on healthy moDC, measured as either percentage of positive cells or MFI, was not affected by continuous ligation of ILT4 or ILT2/ILT4 (Figure 3(b)). Interestingly, in SLE patients ILT2 in synergy with ILT4 seemed to have a slight but evident effect on moDC maturation. The percentage of CD40 and CD80 positive moDC, as well as surface expression level of CD86 and CD83, tends to diminish in the presence of the continuous ligation of ILT2/ILT4 (Figure 3(c)).

### 3.3. Effect of the Activation of ILT4 and/or ILT2 on Cytokine Release by DC from SLE Patients

**Figure 4:**
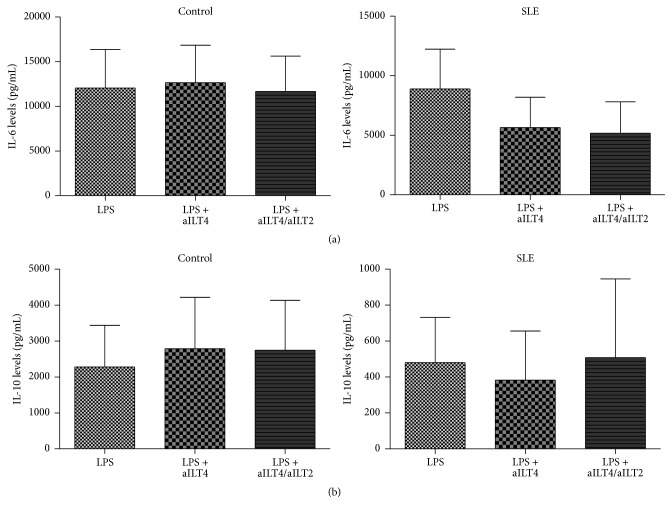
Effect of ILT4 ligation on cytokine release by DC from patients with SLE and healthy controls. DC generated* in vitro* with GM-CSF/IL-4 were maturated for 48 h with LPS or LPS with anti-ILT4 or LPS plus anti-ILT4/anti-ILT2 agonistic mAbs (aILT4/ILT2). Then, culture supernatants were obtained, and the concentrations of the indicated cytokines were determined by flow cytometry. (a) Levels of IL-10 in culture supernatants from healthy controls (left panel) and SLE patients (right panel). (b) Levels of IL-6 in culture supernatants from healthy controls (left panel) and SLE patients (right panel). Results are shown as the median and the interquartile range.

In order to evaluate the possible effect of ILT4 and/or ILT2 signaling in the regulation of cytokine production by differentiated moDC* in vitro*, we quantified cytokine levels by flow cytometry in the cell culture supernatants. DC were generated* in vitro* as described in Section 2 and maturated for 48 h with LPS in the presence or absence of anti-ILT4 and/or anti-ILT2 agonistic mAbs. TNF-*α*, IL-6, IL-4, and IL-10 concentrations were determined in culture supernatants (Tables 2 and 3). Continuous ligation of ILT4 and/or ILT2 did not affect IL-6 production by moDC from controls. In contrast, ILT4 plus ILT2 ligation induced a slight decrease in IL-6 production by moDC from SLE patients (Figure 4(a)). Interestingly, in these patients, IL-10 levels tended to be higher in response to ILT2 and ILT4 engagement (Figure 4(b)).

### 3.4. Role of ILT4 in the Immunogenic Capability of moDC from SLE Patients

**Figure 5:**
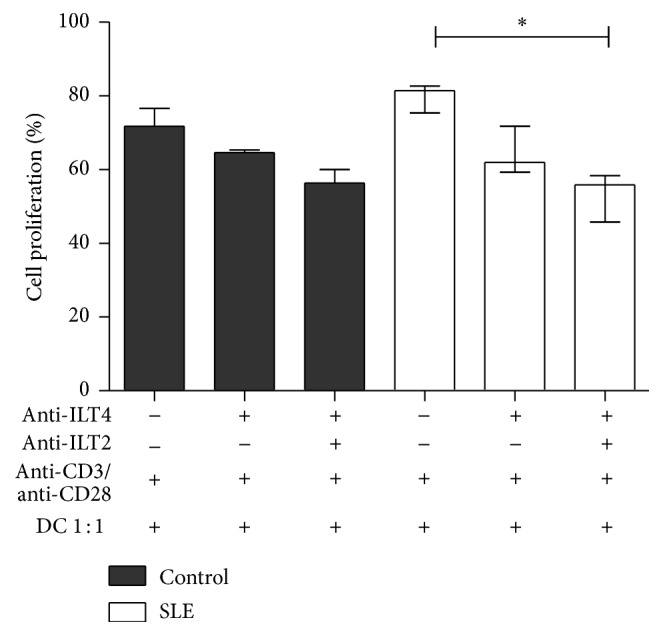
Effect of ILT4 engagement on the immunogenic activity of DC from patients with SLE. Mature DC were incubated in the presence or absence of anti-ILT4 and/or ILT2 agonistic mAb (aILT4/ILT2) and then cocultured with allogenic CFSE labeled PBMC, in flat-bottomed 96-well plates precoated with a mixture of anti-CD3 and anti-CD28 mAb. At day 5, cells were harvested and analyzed by flow cytometry. Percentage of cell proliferation was calculated as follows: % of proliferation = 100 − ((% cells in nonstimulated culture/% cells in stimulated culture) × 100). Empty bars correspond to healthy controls, and filled bars to SLE patients. Median and interquartile range of the data is shown. ^*∗*^ *p* < 0.05.

Finally, in order to assess the role of ILT4 and/or ILT2 in the regulation of immunogenic ability of moDC, we performed cocultures of moDC maturated in the presence or absence of ILT4 and/or ILT2 agonistic mAbs with allogenic PBMC. As mentioned before, ILT4 ligation has been described to confer a lower immunogenic capability on DC [30]. We found that, in SLE patients, ILT4 ligation (alone or in combination with anti-ILT2 mAb) conferred the ability to inhibit PBMC proliferation on moDC, which was not observed in healthy controls (Figure 5).

## 4. Discussion

DC are professional antigen-presenting cells and initiators of the immune response; however, now it is clear that they have a fundamental role in the maintenance of immune tolerance [3]. It had been postulated that immature DC promote tolerogenic responses, whereas mature DC promote immunogenic responses. Recent studies have shown that, under certain circumstances, mature DC can exert a tolerogenic effect [31]. In this regard, it has been reported that the expressions of regulatory receptors belonging to the ILT family, mainly ILT2, ILT3, and ILT4, are associated with a tolerogenic phenotype, inhibiting the expression of costimulatory molecules and triggering IL-10 production [31, 32].

Autoimmune diseases are a consequence of a loss of immune tolerance; it has been described in different animal models that the absence of regulatory receptors that possess inhibitory motifs (ITIMs), including ILT molecules, is associated with autoimmune diseases [10]. Our group has previously reported that T lymphocytes from SLE patients show an abnormal expression and a defective function of the inhibitory receptor ILT2 [23]; even more, we showed that SLE patients have a lower expression of ILT2 on peripheral mDC and pDC compared to healthy controls; however, when we assessed ILT2 function on moDC from SLE patients, we observed that this receptor does not have a critical role in regulating DC maturation [22]. The latter suggests that another receptor may be implicated in this response. In order to assess this possibility, we analyzed the expression and function of another inhibitory receptor of ILT family, ILT4. We found that SLE patients showed lower levels of ILT4 positive circulating pDC and mDC. This diminished expression of ILT4 may contribute to a higher immunogenic phenotype of DC in SLE. Similar to ILT2 expression, we did not find an association of ILT4 expression with disease activity measured by SLEDAI or any current medication, which may suggest an intrinsic alteration in DC rather than a result of the inflammatory milieu observed in SLE patients. It has also been described that monocytes from psoriatic arthritis patients showed a diminished expression of ILT4, which indicates that an alteration of these inhibitory receptors may not be exclusive of SLE patients but a common feature with other autoimmune diseases [21].

We observed that moDC from SLE patients display aberrant responses to a maturation stimulus. In agreement with previous studies, the expression of CD80, CD86, and CD40 after culture with LPS was diminished in moDC from lupus patients compared with healthy controls [29, 33, 34]. Ding et al. in 2006 demonstrated that the expressions of maturation and differentiation markers (CD80, CD86, and HLA-DR) were significantly higher in moDC from SLE patients than in healthy controls in the absence of exogenous maturation stimuli (LPS). They also report that, compared with healthy controls, the upregulation of maturation markers in response to maturation stimuli was blunted in the lupus group [34]. Crispín et al. reported increased levels of CD80 and CD86 expression in peripheral blood DC from patients with SLE, and they demonstrated an impaired response to LPS in moDC from these patients [29]. It is possible that the defective response to LPS of lupus moDC may be due to a preactivation state that makes them refractory to further activation signals. However, an abnormal signaling through TLR4/CD14 in SLE patients cannot be excluded. We found a positive correlation between disease activity and expression of maturation markers. moDC from SLE patients with a SLEDAI index ≥ 8 expressed significant lower levels of CD40 and CD80. It is clear from these results that the inflammatory microenvironment may contribute to this abnormal response observed in SLE patients.

It has been described that ILT receptor inhibits DC maturation [32]; however, our previous results showed that ILT2 does not have great impact on inhibiting the moDC upregulation of costimulatory molecules following a maturation stimulus like LPS. This was the rationale for studying ILT4 function. When we assessed ILT4 role in moDC maturation, we observed that interestingly in healthy controls ILT4 does not inhibit moDC maturation alone nor in combination with ILT2. Nevertheless, in SLE patients, ILT2 and ILT4 showed a slight effect on moDC, inducing a discrete reduction in the expression of costimulatory molecules. We hypothesize that this apparently contradictory result is due to the effect of other inhibitory receptors; thus, in healthy individuals the inhibitory receptors ILT2 and ILT4 could play a secondary role in the generation of tolerance of DC, while other molecules, like PDL-1, OX-40L, and ICOSL, may play a crucial role in inducing that regulatory phenotype; conversely, in SLE patients, the existence of a function impairment of the receptors mentioned above may highlight the inhibitory effect of ILT2 and ILT4 not observed in healthy controls. In this regard, Carvalheiro et al. have found a lower expression of ICOSL mRNA in monocytes and pDC from SLE patients with active disease [35]. Another report shows that monocytes and mDC from SLE patients have lower levels of the inhibitory receptor PDL-1 [36].

It is worth mentioning that in this study we have not assessed the contribution of other ILT family members, such as ILT1, which with their interaction with MHC class I molecules, expressed in DC, may counteract ILT2 and ILT4 function. In previous reports, the authors showed that the simultaneous ligation of ILT4 and ILT2 induces an arrest of maturation of DC [32]; however, these results may be due to the protocol used for the differentiation of monocytes into DC since they added TGF-*β* to the differentiation culture; it is known that TGF-*β* increases the levels of ILT receptors and thus an increased level of ILT expression may allow the appreciation of a greater effect after the ligation of these receptors [36].

In regard to moDC function, it has been demonstrated that moDC from SLE patients induce higher activation and proliferation of allogenic PBMCs than moDC from healthy controls. This result is in agreement with previous reports. Jin et al. found that circulating pDC from SLE had an increased ability to stimulate T cells when compared with control pDC [37]. Ding et al. also showed that lupus DC promote an increased T cell activation and alloproliferation [34].

The continuous ligation of ILT2 and ILT4 has been associated with a lower immunogenic capability of moDC [12, 38]. Silencing of inhibitory ILT expression in APC has been found to increase T cell proliferation and synthesis of proinflammatory cytokines [39]. We observed that either ILT2 or ILT4 signaling does not modify the immunogenic ability of DC from healthy controls; we could only appreciate a slight effect in the inhibition of alloproliferation of lymphocytes from this group. Liang et al. showed that the inhibitory signal through ILT receptors depends on the specific ligand present in the culture. In this study, Liang et al. used different isoforms of the ILT2 and ILT4 ligand, HLA-G. They observed that HLA-G5 dimer and HLA-G1 tetrameric complexes have a high capacity to induce an inhibitory signal and modulation of DC activation and maturation, while HLA-G5 monomer did not trigger an inhibitory signal in DC, concluding that the role of different isoforms of HLA-G depends on their concentration and conformation, the latter affecting binding to a specific receptor [40]. On this basis, under our experimental conditions, ILT4 signaling does not seem to impact DC function but the real impact of this receptor* in vivo* may be difficult to elucidate since the different ligands present in the microenvironment may change easily. However, DC from SLE patients showed a decrement on its immunogenic capability upon ligation with ILT2 and ILT4, which supports the hypothesis that in healthy subjects the control of DC activity may rely on other inhibitory receptors; in contrast, in SLE patients the function of these inhibitory receptors may be impaired, and then ILT4 function is more evident.

DC from SLE patients showed an impaired production of cytokines, mainly IL-10 and IL-6. We did not observe an effect on cytokine production by the ligation of ILT4. It has been reported that medication with chloroquine influences proinflammatory cytokine levels. Chloroquine inhibits the production of IL-6 and IFN-*γ* [41–43]. There is also evidence that levels of IL-10 decreased after corticosteroid and chloroquine treatment [44]. In this respect, it is important to point out that several of the studied patients were under therapy with chloroquine (Table 1), and we cannot exclude the treatment effect on the cytokine levels detected.

In conclusion, we found that in nonpathological conditions ILT4, alone or in synergy with ILT2, does not have a crucial role in regulating maturation and immunogenic function of DC, and these characteristics may possibly rely on other inhibitory receptors, such as PDL-1, OX-40L, or ICOSL. It is feasible that, in SLE patients, defects on these receptors highlight ILT2 and ILT4 function; however, even when the function of ILT4 is preserved in DC from SLE patients, the diminished percentages of ILT4 circulating DC may have a role in SLE pathogenesis.

## Figures and Tables

**Table 1:** Clinical characteristics of systemic lupus erythematosus (SLE) patients.

	SLE	Controls
Sex (F/M)	32/2	32/2
Age (years, mean ± SD)	35.2 ± 2.1	33.6 ± 3.3
Disease duration (years)	4.47 ± 1.3	
Treatment		
Methotrexate	21	
Prednisone	22	
Antimalarials	16	
Mycophenolate	5	
Azathioprine	4	
Cyclosporine	2	
SLEDAI (mean ± SD)	5.9 ± 0.8	
SLEDAI ≥ 8	12	
SLEDAI ≤ 8	20	

Dosage: methotrexate 12.5–20 mg/week; prednisone 5–10 mg/day; chloroquine 150–300 mg/day; azathioprine 100 mg/day; mycophenolate mofetil 2 g/day; and cyclosporine 50–100 mg/day weight adjusted.

**Table 2:** Effect of activation of ILT4 and/or ILT2 on cytokine release by DC from healthy controls.

	LPS	LPS + aILT4	LPS + aILT4/ILT2	*p*
IL-10	2276 ± 2597	2787 ± 3189	2742 ± 3110	0.13
IL-6	12038 ± 9676	12647 ± 9333	11659 ± 8828	0.98
IL-4	18916 ± 2508	19512 ± 1944	18464 ± 1840	0.96
TNF *α*	178.1 ± 0.76	227.8 ± 60	261.8 ± 147	0.2

Cytokines levels are expressed as pg/mL. Data are shown as median ± SD.

**Table 3:** Effect of activation of ILT4 and/or ILT2 on cytokine release by DC from SLE patients.

	LPS	LPS + aILT4	LPS + aILT4/ILT2	*p*
IL-10	480 ± 560	382.7 ± 611.3	507.9 ± 980	0.05
IL-6	8876 ± 7487	6645 ± 7407	5768 ± 6879	0.5
IL-4	19533 ± 2059	19471 ± 2682	19323 ± 2152	0.93
TNF *α*	160 ± 163	106.6 ± 100	93.1 ± 7.28	0.38

Cytokines levels are expressed as pg/mL. Data are shown as median ± SD.
